# KATENA: a verifiable governance architecture for encrypted cloud storage systems

**DOI:** 10.3389/fdata.2026.1829960

**Published:** 2026-05-15

**Authors:** Jesús F. Rodríguez-Aragón, Carolina Zato, Francisco Pinto-Santos, Lorena Sánchez-Pravos

**Affiliations:** 1 School of Engineering and Technology (ESIT), International University of La Rioja (UNIR), Logroño, Spain; 2 Department of Computer Science and Automation, University of Salamanca, Salamanca, Spain; 3 BISITE Research Group, University of Salamanca, Salamanca, Spain; 4 Multidisciplinary University Institute for Business, University of Salamanca, Salamanca, Spain

**Keywords:** client-controlled encryption, cloud security, encrypted cloud storage, hierarchical key management, transparency logs, verifiable governance

## Abstract

Modern data-intensive infrastructures increasingly rely on cloud storage and client-controlled encryption to protect the confidentiality of outsourced information. However, while encryption prevents providers from accessing plaintext data, governance operations such as sharing, revocation, and policy updates typically remain opaque to users and auditors. This creates a structural gap between strong data confidentiality and verifiable governance in cloud environments that manage large volumes of sensitive information. This paper introduces KATENA (Key Architecture for Trustworthy Encrypted Networked Archives), an architectural model that enables client-verifiable governance in encrypted cloud storage systems. The proposed approach combines hierarchical key orchestration, transparency-based governance logging, and cryptographically verifiable governance artifacts so that clients can independently validate governance events without relying on provider-side trust. By integrating accountability mechanisms directly into encrypted storage architectures, the work provides a governance-by-design framework that bridges client-controlled encryption with verifiable data governance in modern data-intensive cloud systems.

## 1 Introduction

Cloud storage platforms have become a fundamental component of modern data infrastructures (Backendal et al., 2024; Hofmann and Truong, 2024), yet the governance of encrypted data stored in these systems remains largely opaque to users and auditors. Organizations increasingly rely on cloud services to store and manage large volumes of information due to their scalability, availability, and operational flexibility. However, outsourcing storage and coordination services to external providers introduces significant concerns regarding confidentiality, trust, and governance transparency in large-scale data ecosystems.

Client-controlled encryption has emerged as an important approach to protect the confidentiality of outsourced data in cloud environments (MEGA Limited, 2022). In these systems, cryptographic operations are performed on the client side, ensuring that cloud providers cannot access plaintext data or encryption keys. While this model significantly strengthens confidentiality guarantees, it does not fully address governance challenges associated with managing encrypted data.

In practice, governance operations such as sharing, revocation, key distribution, and policy enforcement are often coordinated through provider-side services. As a result, users typically have limited visibility into how governance decisions are executed. Even when data confidentiality is cryptographically protected, the correctness of governance operations frequently relies on implicit trust in the cloud provider.

This situation creates a structural gap between strong cryptographic protection and verifiable data governance. Existing research has extensively explored encrypted storage architectures, cryptographic access control mechanisms, and transparency infrastructures (Goh et al., 2003; Kallahalla et al., 2003; Li et al., 2004; Laurie et al., 2013; Melara et al., 2015). However, these approaches typically address confidentiality, key management, or verifiable infrastructures in isolation. As a consequence, encrypted cloud storage systems rarely provide mechanisms that allow clients to independently verify how governance operations are executed.

In particular, existing systems rarely combine client-controlled encryption, scalable hierarchical key management, and transparency-based verification mechanisms that allow governance operations to be independently validated by clients.

A representative example of this limitation arises in collaborative environments where access revocation must be enforced across multiple clients. Consider a scenario in which a user is granted access to an encrypted collection and subsequently revoked. While the revocation operation may be correctly issued by the data owner, other clients interacting with the system may not have a reliable mechanism to verify whether the revocation has been consistently applied. As a result, stale key material or outdated metadata may allow continued access to data that should no longer be accessible. This situation illustrates how, in the absence of verifiable governance mechanisms, the effective access state observed by clients may diverge from the intended policy state.

To address this challenge, this paper introduces *KATENA* (*Key Architecture for Trustworthy Encrypted Networked Archives*), an architectural model designed to enable verifiable governance in encrypted cloud storage systems. KATENA combines client-side key ownership, hierarchical key orchestration, encrypted metadata structures, and transparency-based governance evidence in order to make governance operations independently verifiable by clients.

The central idea behind KATENA is to treat governance operations as verifiable state transitions derived from cryptographically verifiable artifacts recorded in append-only transparency logs. Through this approach, clients can reconstruct and validate governance history without relying on provider-side trust assumptions.

The main contributions of this paper are summarized as follows:

- We introduce **KATENA**, an architectural model that enables verifiable governance in encrypted cloud storage systems through client-side key ownership and transparency-based governance evidence.
- We formalize a governance verification model in which governance operations are represented as verifiable events recorded in append-only transparency logs.
- We define a client-side verification procedure that validates governance artifacts using cryptographic signatures, log inclusion proofs, and consistency proofs.
- We analyze the security properties of the architecture under a realistic threat model, including confidentiality preservation, hierarchical key isolation, governance verifiability, and auditability.

The remainder of the paper is organized as follows. Section 2 reviews relevant research on encrypted cloud storage and verifiable infrastructures. Section 3 introduces the system model and the trust assumptions underlying the proposed architecture. Section 4 presents the KATENA architecture and its main components. Section 5 describes the verifiable governance model implemented by the system. Section 6 defines the threat model considered in this work, while Section 7 analyzes the security properties provided by the architecture. Section 8 discusses overhead and deployment considerations, and Section 9 analyzes design trade-offs and potential extensions.

## 2 Related work and research gap

Research on secure cloud data systems spans several areas, including encrypted storage architectures, secure collaboration frameworks, transparency infrastructures, and cryptographic access control mechanisms. This section summarizes the most relevant work across these domains and positions the KATENA architecture within this research landscape.

### 2.1 Early encrypted storage architectures

Early research on encrypted storage systems explored how cryptographic techniques could be used to protect data stored on untrusted servers. One of the first influential systems was SiRiUS, which introduced a cryptographic overlay enabling users to securely store data on untrusted network storage infrastructures (Goh et al., 2003). The Plutus architecture later proposed scalable mechanisms for secure file sharing in untrusted storage environments using cryptographic key management techniques (Kallahalla et al., 2003). Other work such as SUNDR introduced mechanisms to detect server misbehavior by enforcing fork consistency properties in distributed storage repositories (Li et al., 2004).

These systems established many of the core design principles used in modern encrypted storage architectures, including client-side encryption, cryptographic key management, and the assumption that the storage provider cannot be fully trusted for data confidentiality.

### 2.2 Secure collaboration and encrypted data processing

Subsequent research explored how encrypted storage systems could support collaborative environments and distributed applications. Systems such as Depot aimed to reduce the trust required in cloud storage providers by combining cryptographic protection with distributed consistency mechanisms (Mahajan et al., 2010). Similarly, SPORC introduced mechanisms for secure group collaboration using untrusted cloud services while maintaining confidentiality through client-side cryptographic operations (Feldman et al., 2010).

Other work focused on enabling computation and application logic over encrypted data. The CryptDB system demonstrated how encrypted database queries could be executed while preserving confidentiality guarantees in outsourced infrastructures (Popa et al., 2011). The Mylar platform extended these ideas to web applications, enabling developers to build applications that store and share encrypted data in the cloud while preserving end-to-end confidentiality (Popa et al., 2014). Distributed storage systems such as Tahoe-LAFS further demonstrated how least-authority principles and client-side encryption can be applied in practical storage architectures (Wilcox-O'Hearn and Warner, 2008).

### 2.3 Transparency and verifiable infrastructures

Beyond confidentiality, recent research has explored mechanisms that enable verifiability and accountability in distributed infrastructures. Transparency-based approaches have become an important technique for detecting misbehavior in large-scale systems. Certificate Transparency introduced publicly verifiable append-only logs that allow clients to detect fraudulent certificate issuance in public key infrastructures (Laurie et al., 2013). Similar ideas have been applied to cryptographic key infrastructures, such as the CONIKS system, which enables users to verify the integrity of key directories used in secure messaging systems (Melara et al., 2015).

Subsequent work on key transparency protocols extended these ideas by providing verifiable key distribution infrastructures capable of detecting server misbehavior (Garg and Elahi, 2024). Other proposals such as the Accountable Key Infrastructure (AKI) aim to provide accountability in certificate ecosystems (Kim et al., 2013). Infrastructure frameworks such as Trillian have also been developed to implement general-purpose verifiable data structures and transparency logs that can support security-critical applications (Google, 2016). More recently, optimized key transparency systems such as OPTIKS have further improved the scalability and practical deployment of verifiable key-directory infrastructures, reinforcing the relevance of transparency-based verification in large-scale distributed environments (Len et al., 2024).

### 2.4 Modern encrypted cloud storage and cryptographic access control

More recent research has focused on improving the security, scalability, and governance properties of encrypted cloud storage systems. Backendal, Haller, and Paterson provide a systematic analysis of modern client-controlled encrypted cloud storage architectures and discuss design pitfalls and security assumptions found in deployed systems (Backendal et al., 2024). Empirical studies of real-world encrypted storage platforms have also revealed cryptographic weaknesses and implementation flaws in several commercial providers (Hofmann and Truong, 2024).

Several works have explored advanced cryptographic techniques for secure cloud storage and access control. Proxy re-encryption and key-aggregate encryption mechanisms have been proposed as efficient approaches for managing encrypted data sharing in cloud environments (Chen et al., 2024). Other research proposes multi-layer encryption frameworks that combine distributed storage, encryption, and auditing mechanisms to strengthen privacy guarantees in cloud infrastructures (Mishra et al., 2025).

In parallel, emerging research directions aim to strengthen the trustworthiness of cloud infrastructures through verifiable computing and confidential computing mechanisms. Trusted execution environments and hardware-assisted security mechanisms have been proposed as approaches to protect data and computation even in partially trusted cloud infrastructures. Recent work has also examined trusted execution environments from a security and systems perspective, highlighting both their potential for trust reduction and their practical limitations in adversarial settings (Muñoz et al., 2023). Transparency log systems have also continued to evolve, with recent proposals such as LegoLog exploring configurable transparency infrastructures capable of supporting multiple security-critical applications (Fang et al., 2025).

Commercial encrypted storage platforms have also adopted client-side encryption models to protect user data from the storage provider. For example, the MEGA cloud storage service implements a zero-knowledge architecture in which encryption and key management operations are performed on the client side, ensuring that the provider does not have access to plaintext data or encryption keys (MEGA Limited, 2022; Ji et al., 2022).

### 2.5 Research gap

Despite substantial progress in encrypted storage, cryptographic access control, and transparency infrastructures, these research directions have largely evolved independently. Encrypted cloud storage systems typically provide strong confidentiality guarantees but rely on provider-controlled coordination services for governance operations. Conversely, transparency-based infrastructures provide mechanisms for detecting server misbehavior but are rarely integrated into encrypted data storage architectures.

Consequently, there remains a lack of architectural models that combine client-controlled encryption, scalable hierarchical key management, and transparency-based verification mechanisms in order to make governance operations independently verifiable in encrypted cloud data systems.

The KATENA architecture addresses this gap by integrating hierarchical key orchestration with transparency-based governance evidence, enabling clients to reconstruct and verify governance state without relying on provider-side trust assumptions.

## 3 System model

This section describes the system model underlying the proposed architecture. The system consists of a set of clients interacting with a cloud-based storage infrastructure that provides both data storage and coordination services. Clients are responsible for performing cryptographic operations locally, including encryption, decryption, and key derivation. The cloud provider hosts encrypted data objects, encrypted metadata structures, and governance-related artifacts such as policy manifests and audit logs.

The architecture separates the system into three main domains: the client-side trusted computing base, the control plane, and the data plane. The client side is responsible for protecting cryptographic secrets and executing security-critical operations such as encryption and key management. The control plane manages authentication, governance policies, and key distribution artifacts, while the data plane stores encrypted objects and metadata. This separation allows the cloud infrastructure to provide storage and coordination services without accessing plaintext data or encryption keys.

In the proposed model, the cloud infrastructure is not assumed to be trusted for data confidentiality. The provider may operate the storage services correctly but should not be able to access sensitive information or compromise cryptographic protections. Consequently, the system design ensures that encryption keys remain under the exclusive control of the client environment.

To enable governance verification, the system includes mechanisms that allow clients to validate governance-related operations performed by the cloud provider. These mechanisms rely on verifiable artifacts such as signed policy manifests and append-only transparency logs that record governance events. By validating these artifacts, clients can detect inconsistencies or unauthorized modifications in governance operations.

The system also distinguishes between the data plane and the metadata plane. The data plane stores encrypted content objects, while the metadata plane maintains encrypted structural information such as object identifiers, relationships, and logical organization. Protecting metadata is important because it may reveal sensitive information about stored data even when the content itself remains encrypted (Hofmann and Truong, 2024; Knodel et al., 2024).

### 3.1 Assumptions and trust anchors

KATENA relies on standard cryptographic assumptions, including the security of authenticated encryption and digital signatures. The client environment is assumed to protect long-term secrets (e.g., device keys) against remote adversaries, while acknowledging that full endpoint compromise is out of scope (Section 6). The cloud provider is not trusted for confidentiality and may deviate from the protocol, but cannot break cryptographic primitives.

A key trust anchor is the client's locally stored trusted state, including the latest accepted transparency-log root and policy version. This state enables detection of governance rollback and split-view attacks. Time information (timestamps or monotonic counters) is assumed to be sufficient to establish monotonic evolution of governance states, but KATENA does not require a trusted global clock if monotonic counters are used.

## 4 KATENA architecture

The KATENA architecture introduces a modular framework for encrypted cloud storage that integrates hierarchical key management with verifiable governance mechanisms. The architecture is designed to separate trusted cryptographic operations from untrusted cloud infrastructure while enabling independent verification of governance-related events. This separation allows the cloud provider to offer scalable storage and coordination services without requiring access to sensitive data or encryption keys.

### 4.1 Design goals

The design of the KATENA architecture is guided by a set of security and governance objectives that aim to reconcile strong cryptographic protection with operational transparency in cloud-based storage systems.

#### 4.1.1 G1: client-controlled cryptographic trust anchor

Encryption keys must remain under exclusive control of the client environment and must never be exposed to the cloud infrastructure.

#### 4.1.2 G2: verifiable governance operations

Governance actions such as policy updates, key distribution, sharing operations, and revocations must generate verifiable evidence that can be independently validated by clients.

#### 4.1.3 G3: scalable hierarchical key management

The architecture must support large collections of encrypted objects and collaborative environments through hierarchical key derivation and isolation mechanisms.

#### 4.1.4 G4: provider transparency without data disclosure

The cloud provider should be able to operate storage and coordination services while learning neither plaintext data nor encryption keys.

#### 4.1.5 G5: auditable governance history

All governance operations must produce a tamper-evident record that allows reconstruction and independent verification of the system's governance state.

### 4.2 Design principles

The architecture is guided by three principles. **(i) Key ownership by design:** cryptographic secrets remain client-side and the server stores only encrypted objects and wrapped key artifacts. **(ii) Governance as verifiable evidence:** every governance-relevant operation produces signed artifacts that are recorded in an append-only log to prevent undetectable history manipulation. **(iii) Bounded blast radius:** hierarchical key isolation limits the impact of compromise and enables subtree-scoped rekeying and revocation.

The following subsection describes how these design principles are realized in the concrete components of the KATENA architecture and how responsibilities are divided between the client-side trusted computing base and the cloud infrastructure.

### 4.3 Architectural components and planes

At a high level, the architecture is divided into two main domains: the client-side trusted computing base and the cloud infrastructure. The client side contains the components responsible for cryptographic operations, key management, and governance verification. In contrast, the cloud infrastructure hosts storage services and coordination mechanisms that operate exclusively on encrypted data and governance artifacts.

The client-side trusted computing base includes several components. The KATENA client software provides the cryptographic engine responsible for performing encryption and decryption operations using authenticated encryption primitives. A key orchestration component manages the hierarchical structure of encryption keys used to protect stored objects and collections. In addition, a metadata manager handles encrypted metadata structures that describe the organization of stored data without exposing sensitive information. Finally, a governance verifier module allows the client to validate governance-related events recorded by the cloud infrastructure.

On the server side, the architecture distinguishes between two logical planes. The control plane is responsible for authentication, governance policy management, and storage of key distribution artifacts such as wrapped keys and sharing packages. The data plane stores encrypted content objects and encrypted metadata structures. Because all sensitive information remains encrypted, the cloud provider can operate these services without gaining access to plaintext data.

From an operational perspective, the interaction between client and cloud components follows a structured flow. Clients generate encrypted objects and associated metadata locally and upload them to the data plane, while governance-related artifacts (e.g., policy manifests, key packages, and receipts) are transmitted to the control plane. The cloud infrastructure is responsible for storing and serving these artifacts, but does not interpret their semantic content. Clients retrieve both data and governance evidence and perform all verification steps locally, ensuring that correctness and policy compliance are enforced at the edge of the system rather than within the provider infrastructure.

To support governance verification, the architecture also includes a transparency and audit layer that records governance-related events in append-only logs. These logs may include events such as policy updates, key distribution operations, and revocation procedures. Clients can retrieve and verify these records to ensure that governance operations have been executed consistently and in accordance with system policies.

**Figure 1:**
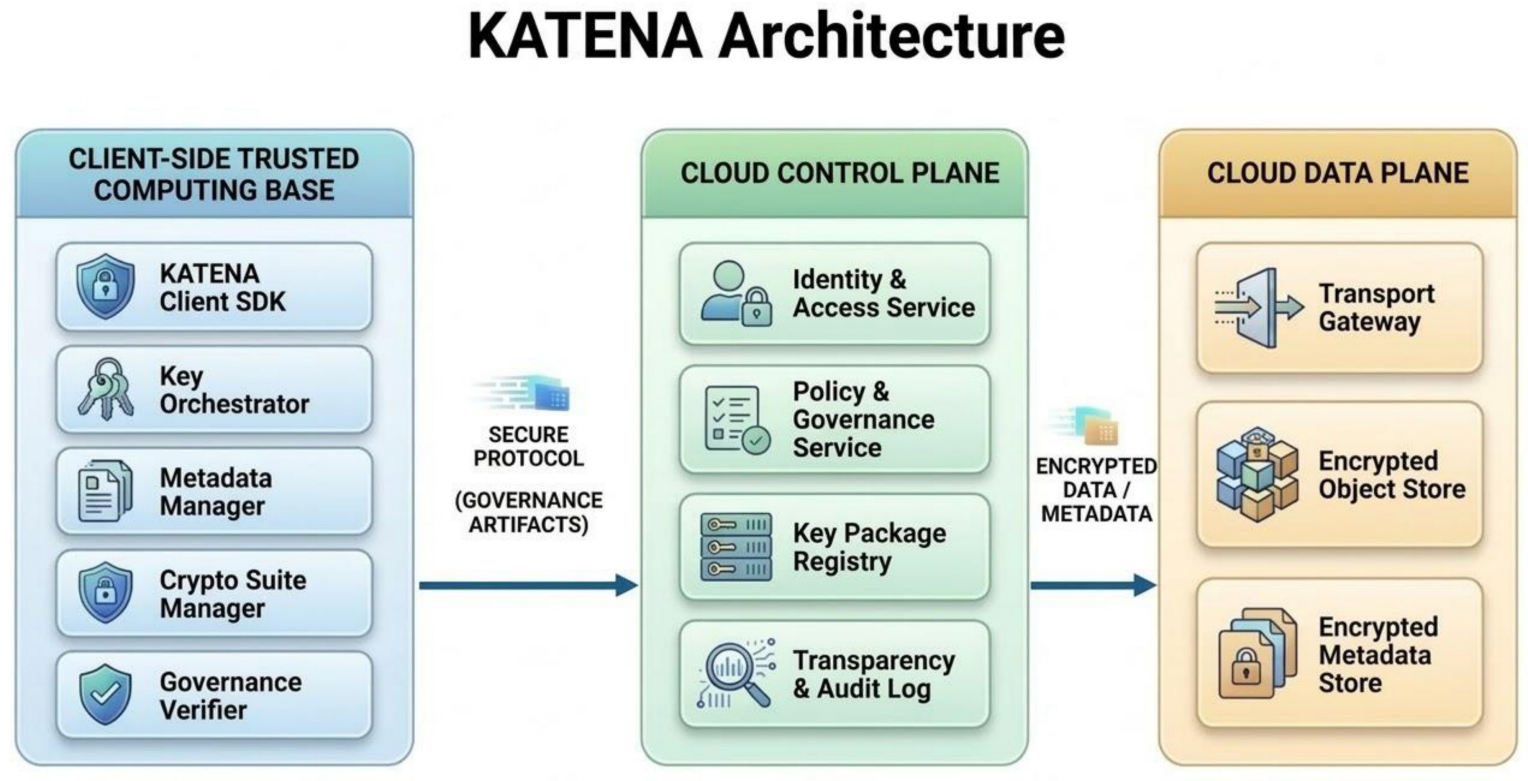
High-level architecture of the KATENA system showing the separation between the client-side trusted computing base and the cloud control and data planes.

Figure 1 illustrates the high-level structure of the KATENA architecture and the interaction between client-side components and the cloud infrastructure.

The architectural components described above establish the structural foundation of the KATENA system. In particular, the separation between the client-side trusted computing base and the cloud infrastructure, together with hierarchical key orchestration and transparency logging, enables governance operations to be expressed as verifiable events. The following section formalizes the governance model that builds on these architectural components.

## 5 Verifiable governance model

Verifiable governance is a central design goal of the KATENA architecture. In the context of encrypted cloud storage systems, governance refers to the mechanisms that regulate how data is accessed, shared, and managed throughout its lifecycle. These mechanisms include operations such as access control enforcement, key distribution, data sharing, policy updates, and revocation procedures.

In many existing cloud storage platforms, governance mechanisms are implemented internally within the provider infrastructure. As a consequence, users often have limited visibility into how governance operations are executed. Even when encryption protects the confidentiality of stored data, the correctness of governance operations typically depends on trusting the provider to enforce policies properly.

The KATENA architecture introduces a verifiable governance model that allows clients to independently validate governance-related operations. Instead of relying solely on provider assurances, governance actions generate cryptographically verifiable artifacts that can be inspected and validated by the client. These artifacts may include signed policy manifests, key distribution records, and log entries describing governance events.

A key component of this model is the use of append-only transparency logs that record governance-related operations. These logs provide an auditable record of events such as policy updates, key sharing operations, and revocations. Because the logs are append-only and cryptographically verifiable, clients can detect inconsistencies or unauthorized modifications in the governance history.

By combining client-side verification with transparency-based logging, the KATENA architecture transforms governance from a trust-based mechanism into a verifiable process. Clients are able to validate that governance operations are consistent with the defined system policies and that no unauthorized actions have been performed by the storage provider.

**Figure 2:**
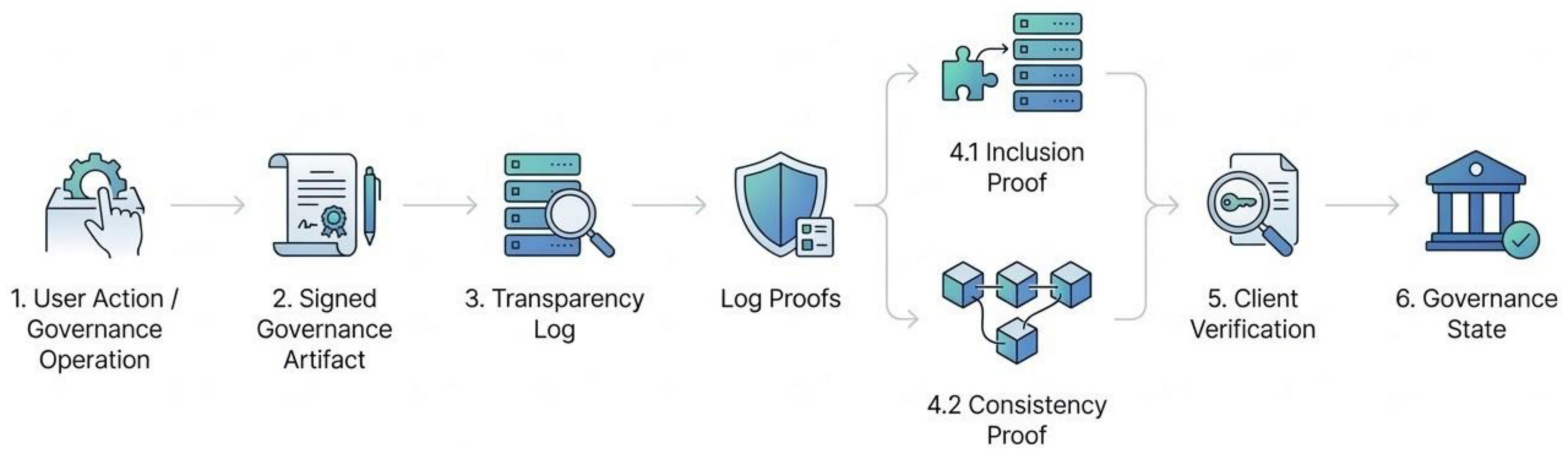
Governance verification pipeline in the KATENA architecture. Governance operations generate signed artifacts that are recorded in an append-only transparency log. Clients retrieve these artifacts, verify log proofs, and reconstruct the resulting governance state.

The overall governance workflow implemented in KATENA can be understood as a pipeline in which governance operations generate signed artifacts that are recorded in transparency logs and later verified by clients. Figure 2 illustrates this governance verification process.

The following subsection formalizes the governance evidence exposed by the cloud infrastructure and the client-side verification procedure.

### 5.1 Governance evidence and verification

To make governance operations independently verifiable, KATENA models governance as a sequence of events recorded in an append-only transparency log. Each governance event produces a signed record and a log commitment that can be validated by clients. This design aims to ensure that a malicious cloud provider cannot undetectably rewrite history, omit governance-relevant events, or present inconsistent views to different clients.

#### 5.1.1 Event model

Let *e*_*i*_ denote the *i*-th governance event for a given tenant, where events include (i) policy publication/update, (ii) key-package publication (e.g., wrapped keys for recipients), and (iii) revocation or rotation announcements. Each event is encoded as *e*_*i*_ = (type,target,payload, *t*_*i*_), where target identifies the affected object/collection (e.g., a node *v* in the key hierarchy), payload contains the governance artifact (e.g., signed policy manifest or key package), and *t*_*i*_ is a timestamp or monotonic counter.

#### 5.1.2 Log commitments

KATENA assumes a log structure that provides append-only properties, e.g., a Merkle-tree-based log. The provider maintains a sequence of log roots {*R*_*i*_}, where *R*_*i*_ commits to the prefix (*e*_1_, …, *e*_*i*_). For each appended event *e*_*i*_, the provider returns a signed receipt σ_*i*_ =Sign_SK_log__(*R*_*i*_, *i*) and an inclusion proof π_*i*_ showing that *e*_*i*_ is included in the tree committed by *R*_*i*_. When a client previously observed *R*_*j*_ and later observes *R*_*k*_ with *k*>*j*, the provider must also return a consistency proof γ_*j*→*k*_ showing that *R*_*k*_ extends *R*_*j*_.

Clients maintain the most recently verified log root and require consistency proofs when observing newer roots. This mechanism prevents equivocation attacks in which a malicious provider attempts to present divergent governance histories to different clients, since inconsistent log views would violate the append-only property of the transparency log.

#### 5.1.3 Verification goals

Given the above evidence, clients verify: (i) authenticity of governance artifacts (signatures on policies and receipts), (ii) log append-only consistency (consistency proofs), and (iii) inclusion of governance events relevant to the client's local state (inclusion proofs). These checks enable detection of governance inconsistencies (e.g., missing revocation events, unauthorized key-package publication, or divergent policy histories).

The correctness of Algorithm 1 follows from the role of each verification step with respect to the attack model. Consistency proof verification ensures that a newly observed log root extends the previously trusted root, thereby preventing rollback and split-view attacks across accepted views. Inclusion-proof verification ensures that the relevant governance events are actually committed under the accepted root, preventing omission or substitution of events. Signature verification on receipts and governance artifacts prevents acceptance of forged evidence. Finally, validation of local governance rules ensures that even correctly logged events are rejected if they violate the client's accepted policy constraints. Taken together, these checks are sufficient to reject the main classes of attacks considered in the threat model for the governance-verification scope of KATENA.

Algorithm 1 **Client-side verification procedure for governance evidence**

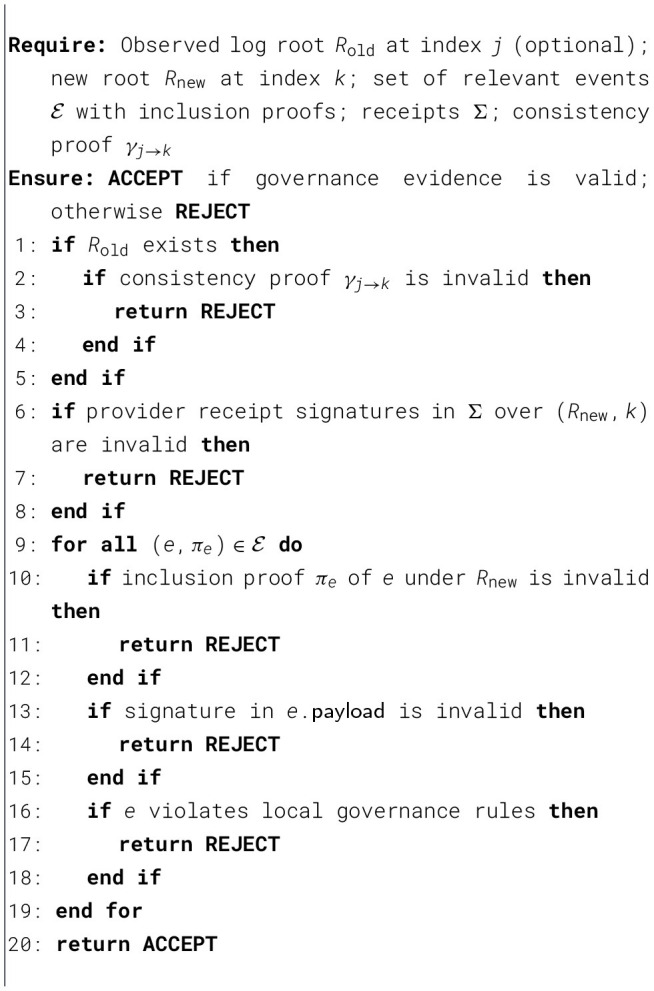

The verification procedure in Algorithm 1 operationalizes the governance guarantees claimed by KATENA. In particular, consistency and inclusion verification directly support **P4 (Governance Verifiability)** by enabling clients to detect split-view and rollback attempts, while the ability to validate and reconstruct governance histories supports **P8 (Auditability)**. These properties are formally defined later in Section 7.1.

The verification procedure follows a fail-fast strategy: any invalid signature, inconsistent log proof, or policy violation immediately causes the client to reject the governance evidence.

#### 5.1.4 Complexity

For Merkle-based transparency logs, inclusion and consistency proofs have logarithmic size and verification cost in the number of log entries *n*. Therefore, validating a batch of *m* governance events relevant to a client requires *O*(*m*log*n*) proof checks, plus signature verification overhead. This bound refers specifically to client-side verification of governance evidence and log consistency. It does not imply that operational update costs such as subtree rekeying, metadata re-encryption, or large-scale revocation are globally bounded by the same expression, since those costs depend on the size of the affected subtree and on the rekeying strategy adopted by the system.

### 5.2 Governance state representation

To reason about the correctness of governance operations, we introduce the notion of a *governance state*. The governance state represents the set of governance rules, key relationships, and policy constraints that determine how encrypted data objects may be accessed and shared within the system.

Formally, the governance state at log index *i* is defined as


$$S_{i}=\left(P_{i},K_{i},R_{i}\right)$$

where *P*_*i*_ represents the set of active governance policies, *K*_*i*_ denotes the current key hierarchy and key distribution relationships, and *R*_*i*_ corresponds to the transparency log commitment representing the prefix of governance events (*e*_1_, …, *e*_*i*_).

Each governance event *e*_*i*_ produces a transition between states such that


$$S_{i}=\delta \left(S_{i-1},e_{i}\right)$$

where δ represents the deterministic governance transition function derived from the policy rules and the event semantics.

Clients verify the consistency of the governance state by validating the authenticity of governance artifacts, checking inclusion and consistency proofs in the transparency log, and ensuring that policy transitions follow the monotonicity constraints defined by the governance rules. This model allows clients to detect unauthorized state transitions or inconsistencies in the governance history presented by the cloud infrastructure.

### 5.3 Governance consistency property

The use of append-only transparency logs allows clients to verify the consistency of the governance state presented by the cloud infrastructure. More precisely, accepted governance states must be derived from validated prefixes of a single append-only event history. Intuitively, a malicious provider should not be able to present two mutually inconsistent governance histories to different clients without detection.

#### 5.3.1 Proposition (governance consistency property)

Let *S*_*i*_ and *S*_*j*_ be two governance states reconstructed by honest clients from validated event prefixes associated with log roots *R*_*i*_ and *R*_*j*_, respectively. If a valid consistency proof exists between *R*_*i*_ and *R*_*j*_, then both states are derived from prefixes of the same append-only governance event sequence. In particular, if *i* ≤ *j*, then the event sequence underlying *S*_*i*_ is a prefix of the event sequence underlying *S*_*j*_.

Proof. By construction, each accepted governance state is obtained by applying the deterministic transition function δ to a validated prefix of governance events recorded in the transparency log. A valid consistency proof between *R*_*i*_ and *R*_*j*_ guarantees that the event sequence committed by one root is a prefix of the event sequence committed by the other, since the log is append-only. Therefore, both accepted states are derived from compatible prefixes of the same governance history rather than from two independent or conflicting histories. Because state reconstruction is deterministic with respect to the validated event sequence, two honest clients accepting log-consistent roots cannot derive mutually inconsistent governance histories unless the append-only property or the proof system itself is violated.

### 5.4 Governance workflows

This subsection describes representative governance workflows in KATENA. The goal is to clarify how key management, policy enforcement, and verification evidence interact in practical operations. For each workflow, the cloud infrastructure remains untrusted for confidentiality, while clients verify governance actions using signed artifacts and transparency-log evidence as described in Section 5.1.

#### 5.4.1 W1: provisioning and onboarding

A user device initializes a local trusted state and binds it to a tenant identity. The client generates (or derives) an initial root governance state and the corresponding cryptographic material used to protect the first-level collections. The cloud provider stores only encrypted artifacts and the initial governance event is appended to the transparency log, enabling other devices to bootstrap by verifying the log root and the signed onboarding artifacts.

#### 5.4.2 W2: secure sharing of a collection

Assume a user shares a collection associated with a hierarchy node *v* with a recipient *u*. The sharing workflow proceeds as follows: (i) the owner client derives or retrieves *K*_*v*_ and produces a wrapped key package Wrap_*u*_(*K*_*v*_) under the recipient's public key (or device key); (ii) the client generates a signed sharing authorization artifact binding (*u, v*) and the key-package identifier; (iii) the cloud stores the key package and the authorization artifact in the control plane; (iv) the event is appended to the transparency log and a receipt is returned; (v) the recipient verifies the receipt, validates inclusion of the event, checks the authorization signature, and only then imports the wrapped key package.

#### 5.4.3 W3: revocation and key rotation

To revoke a user's access to a collection node *v*, the owner triggers a rotation of the affected key material. The client generates a fresh key $K_{v}^{\prime }$ and re-encrypts the minimal required metadata or key-wrapping layer for the affected subtree (depending on the chosen rekeying strategy). A signed revocation/rotation artifact is published and recorded in the transparency log. Clients reject stale sharing packages by checking that the latest log-consistent state includes the revocation event and that local policy versions are monotonic.

#### 5.4.4 W4: policy update and governance rule changes

Governance policies are represented as signed manifests that define authorization rules, acceptable cryptographic suites, and validation constraints (e.g., required proofs for particular operations). A policy update is accepted by clients only if: (i) the manifest signature is valid, (ii) the policy version is monotonic, and (iii) the update event is included in a log root that is consistent with the client's previous root. This prevents rollback and split-view attacks in which different clients are served different policy histories.

## 6 Threat model

The security analysis of the proposed architecture is based on a threat model that captures realistic adversarial capabilities in encrypted cloud storage environments. In this model, the cloud infrastructure is not assumed to be trusted for data confidentiality and may behave as an honest-but-curious or potentially malicious entity. Adversaries may attempt to access encrypted data, manipulate governance artifacts, or interfere with system communications.

**Figure 3:**
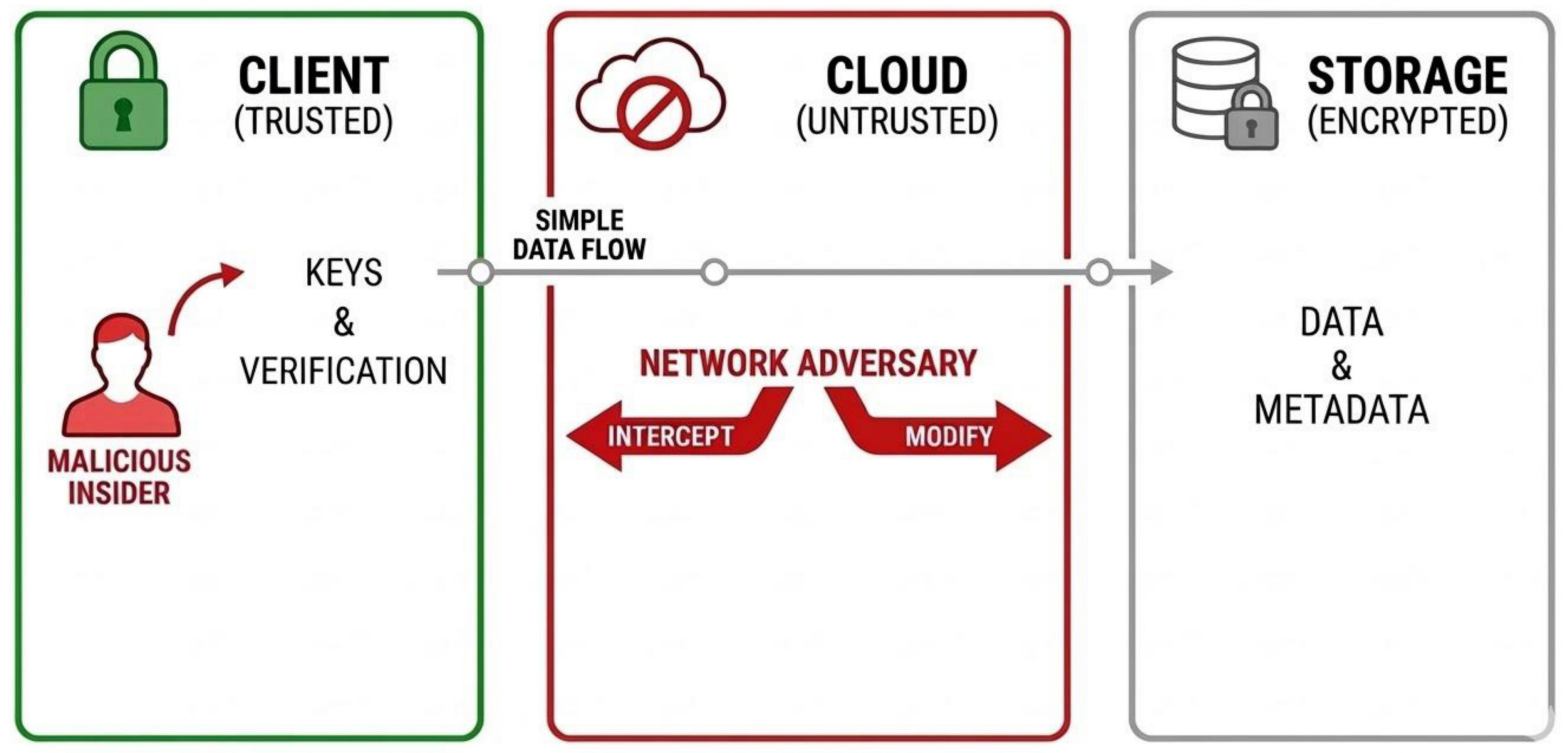
Threat model considered in the KATENA architecture. The client environment is trusted for cryptographic operations and governance verification, while the cloud infrastructure is considered untrusted for confidentiality. Adversaries may control the communication channel or act as malicious insiders with legitimate credentials.

Figure 3 illustrates the main actors and adversarial capabilities considered in the KATENA threat model. The client environment acts as the primary trust anchor of the system, while the cloud infrastructure and the communication channel are considered potentially adversarial.

We consider several classes of adversaries. First, network adversaries may intercept, modify, or replay messages exchanged between clients and the cloud infrastructure. These adversaries are assumed to have full control over the communication channel but cannot break standard cryptographic primitives such as authenticated encryption or digital signatures.

Second, a malicious cloud provider may attempt to analyze stored ciphertexts, manipulate governance artifacts, omit governance events from transparency logs, or present inconsistent system views to different clients. The adversary may also attempt rollback or split-view attacks on the transparency log, presenting divergent log histories to different clients in order to conceal unauthorized governance operations. The architecture therefore assumes that the cloud provider may deviate from the protocol but cannot compromise the underlying cryptographic primitives.

The model also considers insider adversaries with legitimate system credentials. Such actors may attempt to escalate privileges, access unauthorized data, or exploit weaknesses in key distribution mechanisms. Insider threats are particularly relevant in collaborative storage environments where multiple users interact with shared encrypted datasets.

Finally, we consider the possibility of endpoint compromise, in which an attacker gains control of a client device. In this situation, the attacker may obtain access to locally stored keys and decrypted data. While complete endpoint compromise cannot be fully mitigated by the storage architecture itself, the KATENA design aims to limit the impact of such attacks through hierarchical key isolation, revocation mechanisms, and independently verifiable governance records.

Under this threat model, the primary security objectives of the system include protecting data confidentiality, ensuring the integrity of governance artifacts, and enabling clients to detect unauthorized governance operations performed by the cloud infrastructure.

## 7 Security analysis

This section analyzes the security guarantees provided by the KATENA architecture under the threat model described in the previous section. The analysis focuses on several key properties required for secure encrypted cloud storage systems, including data confidentiality, key ownership, governance verifiability, and auditability.

A primary objective of the architecture is to ensure that the confidentiality of stored data does not depend on trusting the cloud provider. In KATENA, encryption operations are performed exclusively on the client side using authenticated encryption primitives. Because encryption keys remain under client control, the cloud infrastructure stores only ciphertext objects and cannot access plaintext data even if the storage system is compromised.

Another important property is client-side key ownership. In the proposed architecture, encryption keys are generated and managed within the client environment and are never exposed in plaintext form to the cloud infrastructure. The server may store wrapped key artifacts used for distribution or sharing, but these artifacts cannot be used to derive encryption keys without the appropriate client-side secrets.

**Figure 4:**
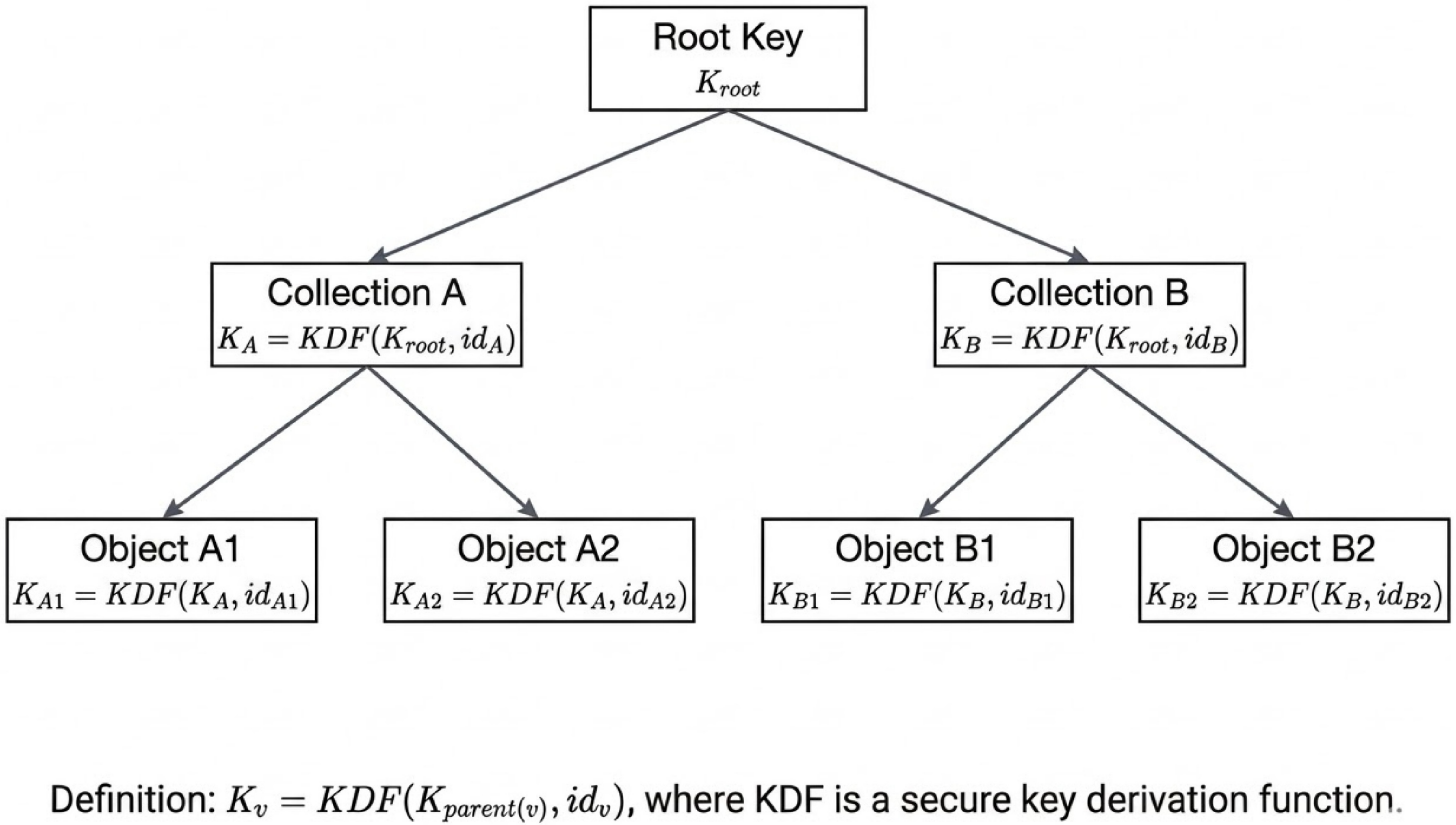
Hierarchical key derivation structure used in the KATENA architecture. Each node key is deterministically derived from its parent key using a key derivation function. This structure enables scalable key management and limits the impact of key compromise to a specific subtree.

KATENA also provides hierarchical key isolation through the use of a structured key hierarchy. As illustrated in Figure 4, encryption keys are organized in a hierarchical structure where higher-level keys protect the keys of subordinate collections and objects. This approach is conceptually related to classical hierarchical cryptographic access control schemes, such as the key derivation framework introduced by (Akl and Taylor, 1983).

Each node in the hierarchy corresponds to a logical collection or object and is protected by a distinct encryption key. If a particular key is compromised, the exposure is limited to the corresponding subtree of objects and does not affect unrelated data stored elsewhere in the hierarchy.

Keys in the KATENA hierarchy are derived using a secure deterministic key-derivation mechanism that binds each node key to its parent key and to a unique node identifier. This design enforces hierarchical isolation by construction: knowledge of a parent key enables derivation of descendant keys within the same subtree, while preventing derivation of keys associated with unrelated branches of the hierarchy.

Formally, the key associated with node *v* is defined as:


$$K_{v}=KDF\left(K_{parent\left(v\right)},id_{v}\right)$$

where *id*_*v*_ uniquely identifies the node in the hierarchy and *KDF* denotes a secure key-derivation function. At the architectural level, the model does not require commitment to a specific instantiation, but assumes a standard derivation mechanism with domain separation through the node identifier to prevent unintended key collisions across the hierarchy. Figure 4 illustrates this derivation structure.

Another security objective is governance verifiability. Governance operations such as policy updates, key distribution events, and revocation procedures generate cryptographically verifiable artifacts recorded in append-only transparency logs. Clients can retrieve and validate these artifacts to ensure that governance operations have been executed consistently and without unauthorized modifications.

The architecture also supports metadata protection. Metadata structures describing the organization of stored data are encrypted to prevent leakage of sensitive information about stored objects. Protecting metadata is important because it may reveal relationships between data items even when the content itself remains encrypted.

Finally, the architecture supports auditable operations through the use of verifiable governance artifacts and transparency logs. These mechanisms allow clients and external auditors to reconstruct the sequence of governance events affecting stored data and to verify that system policies have been enforced correctly.

### 7.1 Security properties

The following analysis is conducted with respect to a probabilistic polynomial-time (PPT) adversary operating under the threat model defined in Section 6. Security properties are expressed in terms of the adversary's ability to break the confidentiality or integrity guarantees of the system.

In addition to confidentiality and key-isolation guarantees, the analysis also characterizes the acceptance guarantees of the client-side verification procedure under the stated trust assumptions.

The security guarantees provided by the KATENA architecture can be summarized through a set of security properties that characterize the behavior of the system under the threat model described in Section 6. These properties capture the main confidentiality and governance guarantees expected from encrypted cloud storage systems.

#### 7.1.1 P1: data confidentiality

Let *M* be a plaintext object and *K*_*v*_ the encryption key associated with node *v* in the key hierarchy. The stored ciphertext is defined as


$$C=Enc\left(K_{v},M\right)$$

where *Enc* is an authenticated encryption function. Without knowledge of *K*_*v*_, an adversary cannot recover *M* from *C*.

Formally, confidentiality can be expressed in terms of the indistinguishability of ciphertexts. For any probabilistic polynomial-time adversary A,


$$|\mathrm{Pr}\left[A\left(Enc\left(K_{v},M_{0}\right)\right)=1\right]-\mathrm{Pr}\left[A\left(Enc\left(K_{v},M_{1}\right)\right)=1\right]|\leq \varepsilon$$

for any two messages *M*_0_, *M*_1_ of equal length, where ε is negligible.

This formulation corresponds to the standard notion of ciphertext indistinguishability under chosen-plaintext attacks and captures the requirement that encrypted data does not reveal information about the underlying plaintext beyond its length.

In the context of KATENA, this property relies on the fact that all encryption operations are performed exclusively on the client side and that encryption keys are never exposed to the cloud provider. As a result, the confidentiality of stored data reduces to the security of the underlying encryption scheme and is independent of the behavior of the storage infrastructure.

#### 7.1.2 P2: client-side key ownership

Encryption keys are generated and maintained exclusively within the client environment. The cloud infrastructure may store wrapped key artifacts but does not obtain direct access to plaintext keys.

#### 7.1.3 P3: hierarchical key isolation

Let *K*_*v*_ be the encryption key associated with node *v* in the hierarchical key structure. Compromise of *K*_*v*_ should not enable an adversary to derive encryption keys associated with unrelated nodes in the hierarchy. In particular, for any node *u* such that *u*≠*v* and *u* is not a descendant of *v*, the probability that an adversary can derive *K*_*u*_ given knowledge of *K*_*v*_ should be negligible. Let A denote a probabilistic adversarial algorithm that takes *K*_*v*_ as input and attempts to derive other keys in the hierarchy. The security requirement can be expressed as:


$$\mathrm{Pr}\left[A\left(K_{v}\right)\to K_{u}\right]\approx 0$$

assuming the security of the underlying key wrapping and key derivation mechanisms.

#### 7.1.4 P4: governance verifiability

Governance operations generate verifiable artifacts recorded in append-only transparency logs. Clients can verify the integrity and consistency of these records to detect unauthorized governance actions.

Formally, if two clients observe log roots *R*_*i*_ and *R*_*j*_ that are cryptographically consistent, then the corresponding governance states must correspond to prefixes of the same event sequence.

#### 7.1.5 P5: verification soundness

If governance artifacts, receipt signatures, inclusion proofs, and consistency proofs are valid with respect to the accepted cryptographic public parameters and trust anchors, then an honest client executing the verification procedure accepts the corresponding governance state.

#### 7.1.6 P6: verification binding

A malicious provider cannot cause an honest client to accept forged governance evidence unless the adversary can either forge a valid digital signature, produce a false inclusion or consistency proof for the append-only log, or otherwise violate the security assumptions of the underlying cryptographic primitives.

#### 7.1.7 P7: metadata protection

Metadata structures describing the organization of stored data are encrypted using the same hierarchical key structure, preventing unauthorized disclosure of structural information.

#### 7.1.8 P8: auditability

The sequence of governance operations affecting stored objects can be reconstructed from transparency logs, enabling independent verification and auditing of system behavior.

## 8 Overhead and practical considerations

This section discusses the expected overhead introduced by verifiable governance in KATENA. The analysis is intentionally technology-agnostic and focuses on the asymptotic costs and the main operational trade-offs.

### 8.1 Verification and storage overhead

Let *n* be the number of governance events recorded in the transparency log and let *m* be the number of events relevant to a given client operation (e.g., sharing a collection, validating a policy update, or processing a revocation). Using Merkle-based transparency structures, inclusion and consistency proofs are *O*(log*n*), so validating *m* relevant events costs *O*(*m*log*n*) plus signature verification. In practice, clients can minimize *m* by caching the latest trusted log root and requesting proofs only for events affecting their local state.

On the provider side, storage overhead grows linearly with the number of governance events and stored key packages. This overhead is dominated by small control-plane objects (signed manifests, receipts, and key packages) rather than the encrypted data plane. Therefore, the additional storage required by verifiable governance is expected to be small relative to the encrypted object store.

For clarity, it is important to distinguish between evidence-verification cost and governance-update cost. The former is dominated by proof verification over the transparency log and scales as *O*(*m*log*n*) for *m* relevant events. The latter depends on the operational scope of the update. For example, revocation affecting a large subtree may require additional key rotation, metadata updates, or re-wrapping operations proportional to the affected portion of the hierarchy. KATENA does not claim a uniform logarithmic bound for such update operations; rather, it claims that the resulting governance evidence remains efficiently verifiable once published.

### 8.2 Summary of evidence and checks

Table 1 summarizes the main governance artifacts, the evidence returned to clients, and the corresponding verification costs.

**Table 1:** Governance artifacts, verifiable evidence, and client-side checks in KATENA.

Operation	Evidence	Client checks	Cost
Policy update	Signed manifest, log receipt	signature + inclusion + consistency	*O*(log*n*)
Sharing	Key package, authorization, receipt	signature + inclusion + rules	*O*(log*n*)
Revocation	Revocation artifact, receipt	signature + inclusion + monotonicity	*O*(log*n*)
Bootstrap	Latest root, consistency proof	receipt signature + consistency	*O*(log*n*)
Audit	Batch of events + proofs	batch verification	*O*(*m*log*n*)

### 8.3 Deployment notes

Two deployment considerations are particularly relevant. First, multi-device usage requires clients to share a compact trusted state (e.g., the latest trusted root and policy version) across devices. This can be achieved by device-to-device secure channels or by storing only encrypted state in the cloud while retaining the trust anchor locally. Second, revocation strategies involve a trade-off between immediate cryptographic invalidation and re-encryption overhead. KATENA supports hierarchical isolation to bound the blast radius of compromise and to enable subtree-scoped rekeying when needed.

## 9 Discussion and practical implications

### 9.1 Representative usage scenarios

To illustrate the practical applicability of the proposed architecture, this subsection outlines two representative scenarios in which verifiable governance mechanisms provide tangible benefits.

#### 9.1.1 Collaborative enterprise storage

Consider an enterprise environment where multiple teams collaborate on shared encrypted datasets stored in a cloud infrastructure. In such settings, governance operations such as granting access to new members, revoking privileges of departing employees, or updating data access policies occur frequently. In traditional encrypted storage systems, these operations are typically managed by centralized services operated by the provider.

With KATENA, governance actions generate verifiable artifacts recorded in transparency logs. When a new employee receives access to a project repository, the sharing operation produces a key package and a signed authorization record that is appended to the governance log. Other team members can independently verify that the access grant was performed according to the organization's policy. Similarly, when an employee leaves the organization, revocation events and key rotations can be verified by all participants, ensuring that no unauthorized access remains active.

#### 9.1.2 Regulated data storage and compliance auditing

Another scenario arises in regulated sectors such as healthcare, finance, or public administration, where organizations must demonstrate that access to sensitive data follows strict governance procedures. Regulatory frameworks often require auditable evidence showing when and how data access permissions were granted, modified, or revoked.

In the KATENA architecture, governance logs provide a tamper-evident history of policy changes and key distribution events. External auditors can validate that governance actions follow the defined policies by verifying signed artifacts and log consistency proofs. Because these records are cryptographically verifiable, the auditing process does not require trusting the cloud provider's internal administrative logs.

### 9.2 Comparative analysis with existing systems

To better position KATENA within the landscape of secure storage and verifiable infrastructure systems, we provide a qualitative comparison with representative design points that are frequently discussed in the literature and in deployed platforms. We consider (i) a client-controlled encrypted cloud storage service (MEGA), (ii) a least-authority distributed storage system (Tahoe-LAFS), (iii) an encrypted query processing architecture (CryptDB), and (iv) a transparency-based key directory infrastructure (CONIKS) (MEGA Limited, 2022; Wilcox-O'Hearn and Warner, 2008; Popa et al., 2011; Melara et al., 2015). The comparison emphasizes governance transparency, key-management structure, and whether clients can independently verify governance-relevant behavior.

Table 2 summarizes the main differences between KATENA and several widely discussed systems, including the MEGA encrypted storage platform, the Tahoe-LAFS distributed storage system, the CryptDB encrypted database architecture, and the CONIKS key transparency infrastructure.

**Table 2:** Comparison of KATENA with selected encrypted storage and verifiable infrastructure systems.

System	Client-side encryption	Hierarchical keys	Transparency logs	Verifiable governance
MEGA	Yes	Partial	No	Limited
Tahoe-LAFS	Yes	Limited	No	No
CryptDB	Partial	No	No	No
CONIKS	N/A	N/A	Yes	Partial
KATENA	Yes	Yes	Yes	Yes

The comparison highlights several distinguishing characteristics of the proposed architecture. Systems such as MEGA and Tahoe-LAFS provide strong confidentiality guarantees through client-side encryption, but governance operations are typically managed internally by the provider or coordination infrastructure. As a result, clients have limited visibility into how governance actions such as sharing or revocation are executed.

CryptDB focuses primarily on enabling queries over encrypted data rather than providing a comprehensive architecture for encrypted storage governance. While it introduces important techniques for confidential data processing, it does not address verifiable governance mechanisms or transparency-based auditing.

CONIKS introduces a transparency-based infrastructure for verifying key directories in secure messaging systems. Although its focus is different from encrypted cloud storage, it demonstrates how append-only logs can be used to detect server misbehavior. KATENA extends similar transparency principles to governance operations in encrypted storage systems.

Overall, the comparison illustrates that KATENA combines several properties that are typically addressed independently in existing systems: client-controlled encryption keys, hierarchical key management, transparency-based verification, and explicit governance auditability.

From a governance perspective, the key distinction is that KATENA treats governance not as an internal provider-side control function but as a *client-verifiable state machine* driven by transparency-log events (Section 5.2). This allows different clients to converge on a consistent governance state while detecting rollback and split-view attempts, bridging confidentiality-oriented encrypted storage with accountability mechanisms inspired by transparency systems.

### 9.3 Design trade-offs

The KATENA architecture illustrates how verifiable governance mechanisms can be integrated into encrypted cloud storage systems without sacrificing the confidentiality guarantees provided by client-controlled encryption. By separating trusted client-side cryptographic components from untrusted cloud infrastructure, the architecture enables users to maintain control over encryption keys while still benefiting from the scalability and availability of cloud storage services.

A key advantage of the proposed approach is that governance operations become observable and verifiable by clients. Instead of relying solely on provider assurances, users can validate governance events through cryptographically verifiable artifacts and transparency logs. This capability increases trust in the system and allows potential inconsistencies or unauthorized actions to be detected.

Beyond these immediate advantages, and more broadly, the KATENA model aligns with emerging trends in cloud security and distributed systems. In particular, the shift toward zero-trust architectures emphasizes the need to reduce implicit trust in infrastructure providers and to move verification capabilities to the client side. Similarly, transparency-based mechanisms are increasingly used to provide accountability in security-critical systems, ranging from certificate infrastructures to secure messaging platforms.

From a governance perspective, KATENA can also support compliance and audit requirements in regulated environments by providing verifiable evidence of policy enforcement and access-control decisions. This suggests that verifiable governance mechanisms may play a broader role in future cloud architectures, bridging the gap between cryptographic data protection and externally auditable system behavior.

However, the introduction of transparency logs and governance artifacts may increase the complexity of the storage infrastructure. Additional storage and processing resources may be required to maintain verifiable governance records and to support verification procedures executed by clients. These overheads must be considered when deploying the architecture in large-scale environments. In practice, most of the additional complexity is concentrated in the control plane and does not significantly affect the throughput of the encrypted data plane.

Another limitation relates to the protection of access patterns and metadata leakage. Although KATENA protects the confidentiality of stored data and encrypts metadata structures, some information may still be inferred from access patterns or system interactions. Addressing these concerns may require integrating additional techniques such as oblivious access mechanisms or privacy-preserving data structures.

Future work may explore the integration of post-quantum cryptographic primitives, advanced metadata protection mechanisms, and large-scale deployment scenarios. Evaluating the performance implications of the architecture in real-world cloud infrastructures is also an important direction for future research.

## 10 Conclusion

This paper presented KATENA, an architectural model designed to enable verifiable governance in encrypted cloud storage systems. The proposed architecture separates trusted client-side cryptographic components from untrusted cloud infrastructure while allowing users to independently verify governance operations through cryptographically verifiable artifacts and transparency mechanisms.

By combining client-side key ownership, hierarchical key management, encrypted metadata structures, and append-only governance logs, KATENA enables users to maintain control over their data while benefiting from the scalability and availability of cloud storage services. The architecture also introduces mechanisms that allow governance operations such as policy updates, key distribution, and revocation events to be verified independently by clients.

The security analysis shows that the architecture provides several important properties for encrypted cloud storage systems, including confidentiality of stored data, client-side key ownership, hierarchical key isolation, governance verifiability, verification soundness, verification binding, metadata protection, and auditable operations. These properties help reduce the level of implicit trust required in cloud providers and improve transparency in governance processes.

Future work may include extending the architecture with additional privacy-preserving mechanisms, evaluating its performance in large-scale deployments, and exploring the integration of emerging cryptographic techniques such as post-quantum encryption.

## Data Availability

The original contributions presented in the study are included in the article/supplementary material, further inquiries can be directed to the corresponding author.
